# Diafenthiuron: 1-*tert*-butyl-3-(2,6-diisopropyl-4-phen­oxy­phen­yl)thio­urea

**DOI:** 10.1107/S1600536814014214

**Published:** 2014-06-25

**Authors:** Youngeun Jeon, Gihaeng Kang, Seonghwa Cho, Tae Ho Kim

**Affiliations:** a Department of Chemistry and Research Institute of Natural Sciences, Gyeongsang National University, Jinju 660-701, Republic of Korea

**Keywords:** crystal structure

## Abstract

The title compound, C_23_H_32_N_2_OS, is a thio­urea-based insecticide. The dihedral angle between the phenyl ring and the diisopropyl benzene ring plane is 73.18 (6)°, while that between the plane of the thio­urea group and the diisopropyl benzene ring is 86.00 (5)°. Disorder was modelled for the S atom and the two methyl C atoms of the isopropyl group over two sets of sites with an occupancy ratio of 0.742 (4):0.258 (4). In the crystal, N—H⋯S hydrogen bonds link adjacent mol­ecules, forming *R*_2_^2^(8) inversion dimers that pack into chains along the *b*-axis direction.

## Related literature   

For information on the toxicity and insecticidal properties of the title compound, see: Ishaaya *et al.* (1993 ▶). For a related structure, see: Zhang *et al.* (2010 ▶). For hydrogen-bond motifs, see: Bernstein *et al.* (1995 ▶).
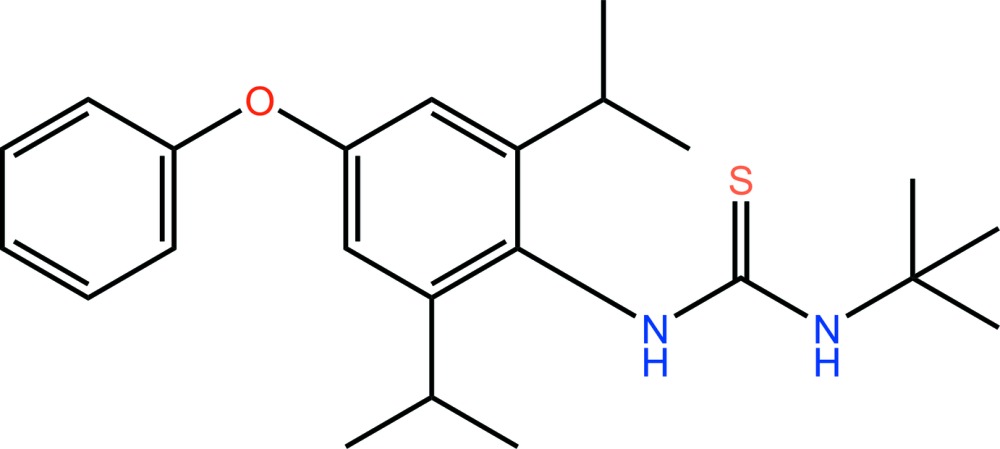


## Experimental   

### 

#### Crystal data  

C_23_H_32_N_2_OS
*M* *_r_* = 384.57
Monoclinic, 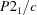

*a* = 12.8656 (2) Å
*b* = 17.9807 (3) Å
*c* = 10.1671 (2) Å
β = 102.655 (1)°
*V* = 2294.84 (7) Å^3^
*Z* = 4
Mo *K*α radiation
μ = 0.16 mm^−1^
*T* = 173 K
0.50 × 0.30 × 0.19 mm

#### Data collection  

Bruker APEXII CCD diffractometer
Absorption correction: multi-scan (*SADABS*; Bruker, 2009 ▶) *T*_min_ = 0.927, *T*_max_ = 0.971
39926 measured reflections
5279 independent reflections
4117 reflections with *I* > 2σ(*I*)
*R*_int_ = 0.034

#### Refinement  

*R*[*F* ^2^ > 2σ(*F* ^2^)] = 0.045
*wR*(*F* ^2^) = 0.119
*S* = 1.03
5279 reflections
281 parameters
19 restraints
H-atom parameters constrained
Δρ_max_ = 0.25 e Å^−3^
Δρ_min_ = −0.23 e Å^−3^

### 

Data collection: *APEX2* (Bruker, 2009 ▶); cell refinement: *SAINT* (Bruker, 2009 ▶); data reduction: *SAINT*; program(s) used to solve structure: *SHELXTL* (Sheldrick, 2008 ▶); program(s) used to refine structure: *SHELXTL*; molecular graphics: *DIAMOND* (Brandenburg, 2010 ▶); software used to prepare material for publication: *SHELXTL*.

## Supplementary Material

Crystal structure: contains datablock(s) global, I. DOI: 10.1107/S1600536814014214/sj5414sup1.cif

Structure factors: contains datablock(s) I. DOI: 10.1107/S1600536814014214/sj5414Isup2.hkl

Supporting information file. DOI: 10.1107/S1600536814014214/sj5414Isup3.cml

CCDC reference: 1008806

Additional supporting information:  crystallographic information; 3D view; checkCIF report

## Figures and Tables

**Table 1:** Hydrogen-bond geometry (Å, °)

*D*—H⋯*A*	*D*—H	H⋯*A*	*D*⋯*A*	*D*—H⋯*A*
N2—H2*N*⋯S1^i^	0.88	2.53	3.3739 (15)	160

Symmetry code: (i) 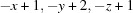.

**Fig. 1.**
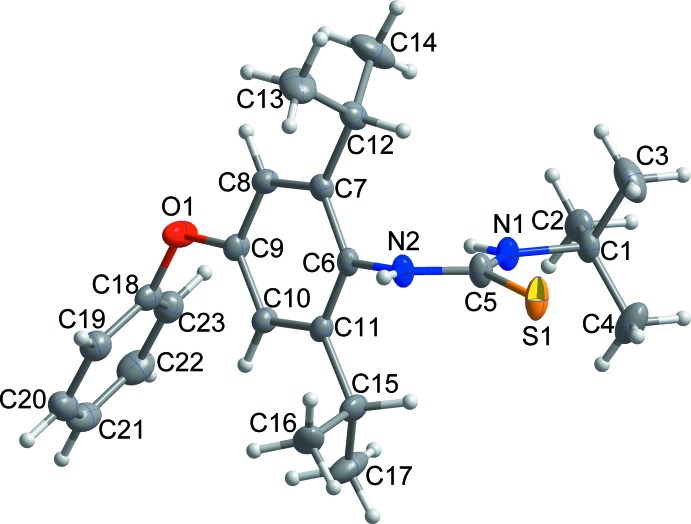
The molecular structure of the title compound. Displacement ellipsoids are drawn at the 30% probability level. H atoms are shown as small spheres of arbitrary radius. Only atoms of the major disorder components are shown.

**Fig. 2.**
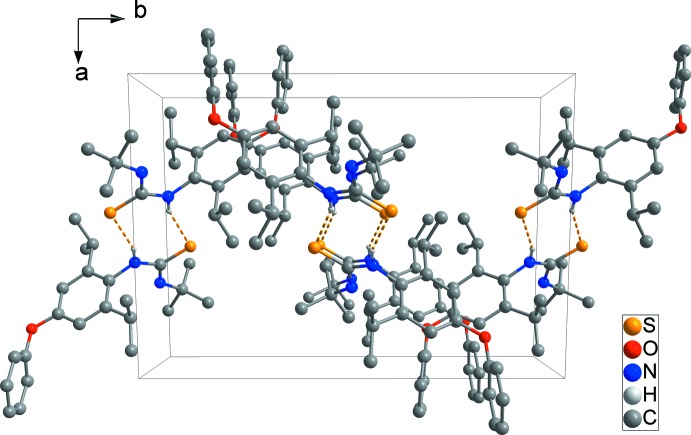
Crystal packing of the title compound with N—H···S hydrogen bonds shown as dashed lines. H atoms bonded to C atoms have been omitted for clarity. Only atoms of the major disorder components are shown.

## supplementary crystallographic information

### S1. Comment

Diafenthiuron (systematic name: 1 - *t*-butyl-3-(2,6-diisopropyl-4-phenoxyphenyl)thiourea), is a type of thiourea insecticide which acts specifically on phytophagous mites, whiteflies and aphids (Ishaaya *et al.*, 1993), and its crystal structure is reported herein. In this compound (Fig. 1), the dihedral angle between the phenyl ring and diisopropyl phenyl ring is 73.18 (6)°. The diheral angle between thiourea group plane and the diisopropyl phenyl is 86.00 (5)°. Disorder was modeled for one S atom (S1) and two C atoms (C16 and C17) of isopropyl group over two sets of sites with an occupancy ratio of 0.742 (4):0.258 (4). All bond lengths and bond angles are normal and comparable to those observed in the crystal structure of a similar compound (Zhang *et al.*, 2010). In the crystal structure (Fig. 2), N2–H2N···S1 hydrogen bonds link adjacent molecules to form R^2^_2_(8) inversion dimers (Bernstein *et al.*, 1995), packed into chains along the *b* axis direction.

### S2. Experimental

The title compound was purchased from the Dr. Ehrenstorfer GmbH Company. Slow evaporation of a solution in CH_2_Cl_2_ gave single crystals suitable for X-ray analysis.

### S3. Refinement

During refinement, one S1 atom and two C16 and C17 atoms of the isopropyl group were found to be disordered and were refined over two sites. The corresponding site-occupation factors were refined so that their sum was unity [0.742 (4) and 0.258 (4)]. All H-atoms were positioned geometrically and refined using a riding model with d(N—H) = 0.88 Å, *U*_iso_ = 1.2*U*_eq_(C) for amine N—H, d(C—H) = 0.95 Å, *U*_iso_ = 1.2*U*_eq_(C) for aromatic C—H, d(C—H) = 0.98 Å, *U*_iso_ = 1.5*U*_eq_(C) for CH_3_ groups, and d(C—H) = 1.00 Å, *U*_iso_ = 1.2*U*_eq_(C) for C*sp*^3^—H.

### Crystal data

**Table:**

C_23_H_32_N_2_OS	*F*(000) = 832
*M**_r_* = 384.57	*D*_x_ = 1.113 Mg m^−^^3^
Monoclinic, *P*2_1_/*c*	Mo *K*α radiation, λ = 0.71073 Å
Hall symbol: -P 2ybc	Cell parameters from 9944 reflections
*a* = 12.8656 (2) Å	θ = 2.6–26.5°
*b* = 17.9807 (3) Å	µ = 0.16 mm^−^^1^
*c* = 10.1671 (2) Å	*T* = 173 K
β = 102.655 (1)°	Block, colourless
*V* = 2294.84 (7) Å^3^	0.50 × 0.30 × 0.19 mm
*Z* = 4	

### Data collection

**Table:**

Bruker APEXII CCD diffractometer	5279 independent reflections
Radiation source: fine-focus sealed tube	4117 reflections with *I* > 2σ(*I*)
Graphite monochromator	*R*_int_ = 0.034
φ and ω scans	θ_max_ = 27.5°, θ_min_ = 1.6°
Absorption correction: multi-scan (*SADABS*; Bruker, 2009)	*h* = −16→16
*T*_min_ = 0.927, *T*_max_ = 0.971	*k* = −23→22
39926 measured reflections	*l* = −13→13

### Refinement

**Table:**

Refinement on *F*^2^	Primary atom site location: structure-invariant direct methods
Least-squares matrix: full	Secondary atom site location: difference Fourier map
*R*[*F*^2^ > 2σ(*F*^2^)] = 0.045	Hydrogen site location: inferred from neighbouring sites
*wR*(*F*^2^) = 0.119	H-atom parameters constrained
*S* = 1.03	*w* = 1/[σ^2^(*F*_o_^2^) + (0.0479*P*)^2^ + 0.7379*P*] where *P* = (*F*_o_^2^ + 2*F*_c_^2^)/3
5279 reflections	(Δ/σ)_max_ = 0.001
281 parameters	Δρ_max_ = 0.25 e Å^−^^3^
19 restraints	Δρ_min_ = −0.23 e Å^−^^3^

### Special details

**Table:**

Geometry. All e.s.d.'s (except the e.s.d. in the dihedral angle between two l.s. planes) are estimated using the full covariance matrix. The cell e.s.d.'s are taken into account individually in the estimation of e.s.d.'s in distances, angles and torsion angles; correlations between e.s.d.'s in cell parameters are only used when they are defined by crystal symmetry. An approximate (isotropic) treatment of cell e.s.d.'s is used for estimating e.s.d.'s involving l.s. planes.
Refinement. Refinement of *F*^2^ against ALL reflections. The weighted *R*-factor *wR* and goodness of fit *S* are based on *F*^2^, conventional *R*-factors *R* are based on *F*, with *F* set to zero for negative *F*^2^. The threshold expression of *F*^2^ > σ(*F*^2^) is used only for calculating *R*-factors(gt) *etc*. and is not relevant to the choice of reflections for refinement. *R*-factors based on *F*^2^ are statistically about twice as large as those based on *F*, and *R*- factors based on ALL data will be even larger.

### Fractional atomic coordinates and isotropic or equivalent isotropic displacement parameters (Å^2^)

**Table:**

	*x*	*y*	*z*	*U*_iso_*/*U*_eq_	Occ. (<1)
S1	0.56002 (13)	1.08803 (6)	0.37193 (18)	0.0552 (3)	0.742 (4)
S1'	0.5179 (4)	1.0709 (2)	0.3222 (4)	0.0580 (10)	0.258 (4)
O1	0.85776 (9)	0.69041 (6)	0.51145 (12)	0.0536 (3)	
N1	0.69688 (10)	1.00011 (7)	0.27909 (12)	0.0403 (3)	
H1N	0.7318	0.9577	0.2897	0.048*	
N2	0.61785 (9)	0.95071 (6)	0.43823 (12)	0.0369 (3)	
H2N	0.5680	0.9532	0.4851	0.044*	
C1	0.72543 (12)	1.05027 (8)	0.17740 (15)	0.0419 (3)	
C2	0.80932 (13)	1.00766 (9)	0.12308 (17)	0.0482 (4)	
H2A	0.8690	0.9951	0.1976	0.072*	
H2B	0.8350	1.0386	0.0575	0.072*	
H2C	0.7779	0.9619	0.0792	0.072*	
C3	0.62948 (15)	1.06426 (14)	0.0633 (2)	0.0745 (6)	
H3A	0.6029	1.0168	0.0218	0.112*	
H3B	0.6503	1.0963	−0.0045	0.112*	
H3C	0.5734	1.0887	0.0989	0.112*	
C4	0.77343 (18)	1.12213 (10)	0.2428 (2)	0.0714 (6)	
H4A	0.7195	1.1494	0.2781	0.107*	
H4B	0.7977	1.1527	0.1757	0.107*	
H4C	0.8340	1.1105	0.3168	0.107*	
C5	0.62684 (12)	1.00853 (8)	0.35760 (15)	0.0426 (4)	
C6	0.68322 (10)	0.88509 (7)	0.45404 (13)	0.0315 (3)	
C7	0.65135 (10)	0.82379 (7)	0.37036 (13)	0.0328 (3)	
C8	0.71330 (11)	0.75966 (8)	0.39362 (14)	0.0358 (3)	
H8	0.6936	0.7172	0.3382	0.043*	
C9	0.80322 (11)	0.75739 (7)	0.49667 (15)	0.0362 (3)	
C10	0.83576 (11)	0.81891 (7)	0.57626 (15)	0.0364 (3)	
H10	0.8988	0.8168	0.6452	0.044*	
C11	0.77584 (11)	0.88429 (7)	0.55526 (14)	0.0344 (3)	
C12	0.55233 (12)	0.82694 (9)	0.25731 (15)	0.0431 (4)	
H12	0.5328	0.8805	0.2404	0.052*	
C13	0.45888 (14)	0.78824 (13)	0.2968 (2)	0.0728 (6)	
H13A	0.4768	0.7359	0.3174	0.109*	
H13B	0.3963	0.7912	0.2221	0.109*	
H13C	0.4432	0.8125	0.3765	0.109*	
C14	0.57182 (19)	0.79454 (14)	0.12726 (19)	0.0823 (7)	
H14A	0.6319	0.8203	0.1025	0.123*	
H14B	0.5079	0.8009	0.0554	0.123*	
H14C	0.5882	0.7414	0.1398	0.123*	
C15	0.81007 (14)	0.95275 (8)	0.64085 (18)	0.0516 (4)	
H15A	0.7900	0.9976	0.5826	0.062*	0.742 (4)
H15B	0.7556	0.9932	0.6208	0.062*	0.258 (4)
C16	0.7452 (3)	0.95541 (14)	0.7595 (2)	0.0611 (8)	0.742 (4)
H16A	0.7684	0.9144	0.8225	0.092*	0.742 (4)
H16B	0.6688	0.9506	0.7205	0.092*	0.742 (4)
H16C	0.7590	1.0029	0.8076	0.092*	0.742 (4)
C17	0.9244 (2)	0.95782 (14)	0.7033 (4)	0.0769 (12)	0.742 (4)
H17A	0.9389	1.0051	0.7519	0.115*	0.742 (4)
H17B	0.9658	0.9553	0.6332	0.115*	0.742 (4)
H17C	0.9447	0.9165	0.7665	0.115*	0.742 (4)
C16'	0.8471 (10)	0.9434 (5)	0.7755 (9)	0.090 (3)	0.258 (4)
H16D	0.9083	0.9096	0.7912	0.136*	0.258 (4)
H16E	0.7906	0.9222	0.8149	0.136*	0.258 (4)
H16F	0.8690	0.9916	0.8174	0.136*	0.258 (4)
C17'	0.9296 (6)	0.9760 (4)	0.5818 (9)	0.062 (2)	0.258 (4)
H17D	0.9606	1.0219	0.6254	0.093*	0.258 (4)
H17E	0.9114	0.9830	0.4839	0.093*	0.258 (4)
H17F	0.9814	0.9354	0.6043	0.093*	0.258 (4)
C18	0.95008 (11)	0.68370 (7)	0.61207 (16)	0.0391 (3)	
C19	0.94465 (13)	0.67609 (9)	0.74495 (17)	0.0478 (4)	
H19	0.8778	0.6774	0.7702	0.057*	
C20	1.03768 (15)	0.66652 (10)	0.84113 (18)	0.0566 (4)	
H20	1.0349	0.6615	0.9333	0.068*	
C21	1.13450 (14)	0.66421 (9)	0.8046 (2)	0.0599 (5)	
H21	1.1983	0.6581	0.8713	0.072*	
C22	1.13828 (13)	0.67082 (10)	0.6715 (2)	0.0610 (5)	
H22	1.2050	0.6687	0.6460	0.073*	
C23	1.04608 (13)	0.68058 (9)	0.57400 (18)	0.0494 (4)	
H23	1.0490	0.6851	0.4818	0.059*	

### Atomic displacement parameters (Å^2^)

**Table:**

	*U*^11^	*U*^22^	*U*^33^	*U*^12^	*U*^13^	*U*^23^
S1	0.0692 (7)	0.0346 (4)	0.0751 (8)	0.0234 (4)	0.0446 (6)	0.0193 (4)
S1'	0.074 (2)	0.0464 (16)	0.0643 (19)	0.0307 (14)	0.0377 (16)	0.0220 (13)
O1	0.0517 (6)	0.0351 (6)	0.0629 (7)	0.0185 (5)	−0.0116 (5)	−0.0140 (5)
N1	0.0508 (7)	0.0319 (6)	0.0442 (7)	0.0112 (5)	0.0236 (6)	0.0083 (5)
N2	0.0453 (6)	0.0309 (6)	0.0405 (7)	0.0131 (5)	0.0223 (5)	0.0078 (5)
C1	0.0491 (8)	0.0382 (8)	0.0434 (8)	0.0027 (6)	0.0212 (7)	0.0101 (6)
C2	0.0540 (9)	0.0492 (9)	0.0481 (9)	−0.0023 (7)	0.0256 (8)	0.0016 (7)
C3	0.0602 (11)	0.1049 (17)	0.0611 (12)	0.0191 (11)	0.0191 (10)	0.0378 (12)
C4	0.0924 (14)	0.0408 (10)	0.0954 (15)	−0.0085 (9)	0.0518 (13)	−0.0068 (10)
C5	0.0529 (8)	0.0347 (7)	0.0465 (8)	0.0136 (6)	0.0244 (7)	0.0094 (6)
C6	0.0362 (7)	0.0275 (6)	0.0344 (7)	0.0069 (5)	0.0155 (6)	0.0044 (5)
C7	0.0336 (6)	0.0339 (7)	0.0327 (7)	0.0048 (5)	0.0109 (6)	0.0033 (6)
C8	0.0388 (7)	0.0316 (7)	0.0365 (7)	0.0035 (5)	0.0069 (6)	−0.0048 (6)
C9	0.0371 (7)	0.0282 (7)	0.0433 (8)	0.0073 (5)	0.0087 (6)	−0.0024 (6)
C10	0.0355 (7)	0.0296 (7)	0.0420 (8)	0.0021 (5)	0.0040 (6)	−0.0006 (6)
C11	0.0398 (7)	0.0260 (6)	0.0386 (7)	0.0003 (5)	0.0113 (6)	0.0001 (6)
C12	0.0435 (8)	0.0433 (8)	0.0394 (8)	0.0100 (6)	0.0025 (6)	0.0031 (7)
C13	0.0411 (9)	0.0935 (16)	0.0742 (14)	−0.0062 (9)	−0.0082 (9)	0.0153 (12)
C14	0.0935 (15)	0.1036 (17)	0.0394 (10)	0.0422 (13)	−0.0080 (10)	−0.0099 (11)
C15	0.0663 (10)	0.0259 (7)	0.0555 (10)	0.0003 (7)	−0.0021 (8)	−0.0046 (7)
C16	0.100 (2)	0.0433 (13)	0.0420 (13)	−0.0090 (13)	0.0198 (14)	−0.0127 (10)
C17	0.0589 (15)	0.0409 (14)	0.118 (3)	−0.0077 (11)	−0.0098 (17)	−0.0191 (16)
C16'	0.162 (10)	0.055 (5)	0.056 (5)	−0.022 (6)	0.029 (6)	−0.020 (4)
C17'	0.061 (4)	0.047 (4)	0.075 (5)	−0.018 (3)	0.006 (4)	−0.019 (4)
C18	0.0390 (7)	0.0250 (6)	0.0501 (9)	0.0080 (5)	0.0025 (6)	−0.0045 (6)
C19	0.0459 (8)	0.0450 (9)	0.0539 (10)	0.0050 (7)	0.0140 (7)	−0.0062 (7)
C20	0.0714 (12)	0.0469 (10)	0.0464 (10)	0.0068 (8)	0.0018 (9)	−0.0007 (8)
C21	0.0484 (9)	0.0441 (9)	0.0749 (13)	0.0078 (7)	−0.0133 (9)	−0.0070 (9)
C22	0.0383 (8)	0.0556 (11)	0.0893 (15)	0.0021 (7)	0.0145 (9)	−0.0087 (10)
C23	0.0524 (9)	0.0428 (9)	0.0558 (10)	0.0033 (7)	0.0176 (8)	−0.0010 (7)

### Geometric parameters (Å, º)

**Table:**

S1—C5	1.6906 (17)	C13—H13A	0.9800
S1'—C5	1.769 (3)	C13—H13B	0.9800
O1—C9	1.3853 (16)	C13—H13C	0.9800
O1—C18	1.3927 (17)	C14—H14A	0.9800
N1—C5	1.3370 (18)	C14—H14B	0.9800
N1—C1	1.4776 (18)	C14—H14C	0.9800
N1—H1N	0.8800	C15—C16'	1.357 (9)
N2—C5	1.3446 (18)	C15—C17	1.472 (3)
N2—C6	1.4373 (16)	C15—C16	1.610 (3)
N2—H2N	0.8800	C15—C17'	1.818 (8)
C1—C3	1.519 (2)	C15—H15A	1.0000
C1—C4	1.521 (2)	C15—H15B	1.0000
C1—C2	1.523 (2)	C16—H16A	0.9800
C2—H2A	0.9800	C16—H16B	0.9800
C2—H2B	0.9800	C16—H16C	0.9800
C2—H2C	0.9800	C17—H17A	0.9800
C3—H3A	0.9800	C17—H17B	0.9800
C3—H3B	0.9800	C17—H17C	0.9800
C3—H3C	0.9800	C16'—H16D	0.9800
C4—H4A	0.9800	C16'—H16E	0.9800
C4—H4B	0.9800	C16'—H16F	0.9800
C4—H4C	0.9800	C17'—H17D	0.9800
C6—C11	1.3935 (19)	C17'—H17E	0.9800
C6—C7	1.3979 (19)	C17'—H17F	0.9800
C7—C8	1.3923 (18)	C18—C23	1.374 (2)
C7—C12	1.5184 (19)	C18—C19	1.375 (2)
C8—C9	1.3809 (19)	C19—C20	1.380 (2)
C8—H8	0.9500	C19—H19	0.9500
C9—C10	1.3802 (19)	C20—C21	1.376 (3)
C10—C11	1.3963 (18)	C20—H20	0.9500
C10—H10	0.9500	C21—C22	1.370 (3)
C11—C15	1.516 (2)	C21—H21	0.9500
C12—C14	1.515 (2)	C22—C23	1.380 (2)
C12—C13	1.518 (2)	C22—H22	0.9500
C12—H12	1.0000	C23—H23	0.9500
			
C9—O1—C18	119.03 (11)	H13A—C13—H13C	109.5
C5—N1—C1	130.69 (12)	H13B—C13—H13C	109.5
C5—N1—H1N	114.7	C12—C14—H14A	109.5
C1—N1—H1N	114.7	C12—C14—H14B	109.5
C5—N2—C6	125.21 (11)	H14A—C14—H14B	109.5
C5—N2—H2N	117.4	C12—C14—H14C	109.5
C6—N2—H2N	117.4	H14A—C14—H14C	109.5
N1—C1—C3	110.53 (13)	H14B—C14—H14C	109.5
N1—C1—C4	110.60 (14)	C16'—C15—C17	58.4 (5)
C3—C1—C4	112.18 (16)	C16'—C15—C11	118.1 (4)
N1—C1—C2	104.55 (12)	C17—C15—C11	115.80 (16)
C3—C1—C2	109.17 (14)	C16'—C15—C16	51.2 (5)
C4—C1—C2	109.51 (14)	C17—C15—C16	107.9 (2)
C1—C2—H2A	109.5	C11—C15—C16	108.72 (14)
C1—C2—H2B	109.5	C16'—C15—C17'	103.1 (5)
H2A—C2—H2B	109.5	C17—C15—C17'	44.8 (3)
C1—C2—H2C	109.5	C11—C15—C17'	99.0 (3)
H2A—C2—H2C	109.5	C16—C15—C17'	148.8 (3)
H2B—C2—H2C	109.5	C16'—C15—H15A	133.2
C1—C3—H3A	109.5	C17—C15—H15A	108.1
C1—C3—H3B	109.5	C11—C15—H15A	108.1
H3A—C3—H3B	109.5	C16—C15—H15A	108.1
C1—C3—H3C	109.5	C17'—C15—H15A	75.4
H3A—C3—H3C	109.5	C16'—C15—H15B	111.8
H3B—C3—H3C	109.5	C17—C15—H15B	129.2
C1—C4—H4A	109.5	C11—C15—H15B	111.8
C1—C4—H4B	109.5	C16—C15—H15B	71.1
H4A—C4—H4B	109.5	C17'—C15—H15B	111.8
C1—C4—H4C	109.5	H15A—C15—H15B	38.1
H4A—C4—H4C	109.5	C15—C16—H16A	109.5
H4B—C4—H4C	109.5	C15—C16—H16B	109.5
N1—C5—N2	115.83 (12)	C15—C16—H16C	109.5
N1—C5—S1	124.69 (11)	C15—C17—H17A	109.5
N2—C5—S1	119.21 (11)	C15—C17—H17B	109.5
N1—C5—S1'	123.91 (15)	C15—C17—H17C	109.5
N2—C5—S1'	116.37 (16)	C15—C16'—H16D	109.5
S1—C5—S1'	24.00 (14)	C15—C16'—H16E	109.5
C11—C6—C7	122.05 (11)	H16D—C16'—H16E	109.5
C11—C6—N2	118.41 (12)	C15—C16'—H16F	109.5
C7—C6—N2	119.52 (12)	H16D—C16'—H16F	109.5
C8—C7—C6	117.97 (12)	H16E—C16'—H16F	109.5
C8—C7—C12	120.90 (12)	C15—C17'—H17D	109.5
C6—C7—C12	121.13 (12)	C15—C17'—H17E	109.5
C9—C8—C7	120.44 (13)	H17D—C17'—H17E	109.5
C9—C8—H8	119.8	C15—C17'—H17F	109.5
C7—C8—H8	119.8	H17D—C17'—H17F	109.5
C10—C9—C8	121.15 (12)	H17E—C17'—H17F	109.5
C10—C9—O1	123.49 (12)	C23—C18—C19	121.01 (14)
C8—C9—O1	115.35 (12)	C23—C18—O1	118.12 (15)
C9—C10—C11	119.91 (13)	C19—C18—O1	120.77 (14)
C9—C10—H10	120.0	C18—C19—C20	119.05 (16)
C11—C10—H10	120.0	C18—C19—H19	120.5
C6—C11—C10	118.41 (12)	C20—C19—H19	120.5
C6—C11—C15	120.62 (12)	C21—C20—C19	120.54 (17)
C10—C11—C15	120.97 (13)	C21—C20—H20	119.7
C14—C12—C13	110.29 (18)	C19—C20—H20	119.7
C14—C12—C7	111.99 (13)	C22—C21—C20	119.62 (16)
C13—C12—C7	111.38 (13)	C22—C21—H21	120.2
C14—C12—H12	107.7	C20—C21—H21	120.2
C13—C12—H12	107.7	C21—C22—C23	120.63 (17)
C7—C12—H12	107.7	C21—C22—H22	119.7
C12—C13—H13A	109.5	C23—C22—H22	119.7
C12—C13—H13B	109.5	C18—C23—C22	119.15 (17)
H13A—C13—H13B	109.5	C18—C23—H23	120.4
C12—C13—H13C	109.5	C22—C23—H23	120.4
			
C5—N1—C1—C3	−62.1 (2)	N2—C6—C11—C15	−3.65 (19)
C5—N1—C1—C4	62.8 (2)	C9—C10—C11—C6	0.6 (2)
C5—N1—C1—C2	−179.42 (16)	C9—C10—C11—C15	−179.73 (14)
C1—N1—C5—N2	178.36 (15)	C8—C7—C12—C14	−45.2 (2)
C1—N1—C5—S1	−7.7 (3)	C6—C7—C12—C14	134.69 (17)
C1—N1—C5—S1'	21.4 (3)	C8—C7—C12—C13	78.84 (19)
C6—N2—C5—N1	5.7 (2)	C6—C7—C12—C13	−101.27 (17)
C6—N2—C5—S1	−168.61 (14)	C6—C11—C15—C16'	137.5 (6)
C6—N2—C5—S1'	164.4 (2)	C10—C11—C15—C16'	−42.1 (6)
C5—N2—C6—C11	90.80 (18)	C6—C11—C15—C17	−156.1 (2)
C5—N2—C6—C7	−90.60 (18)	C10—C11—C15—C17	24.3 (3)
C11—C6—C7—C8	2.2 (2)	C6—C11—C15—C16	82.22 (19)
N2—C6—C7—C8	−176.32 (12)	C10—C11—C15—C16	−97.40 (19)
C11—C6—C7—C12	−177.67 (13)	C6—C11—C15—C17'	−112.3 (3)
N2—C6—C7—C12	3.78 (19)	C10—C11—C15—C17'	68.1 (3)
C6—C7—C8—C9	0.1 (2)	C9—O1—C18—C23	−108.24 (16)
C12—C7—C8—C9	179.97 (13)	C9—O1—C18—C19	75.44 (18)
C7—C8—C9—C10	−2.0 (2)	C23—C18—C19—C20	1.2 (2)
C7—C8—C9—O1	179.17 (13)	O1—C18—C19—C20	177.37 (14)
C18—O1—C9—C10	0.5 (2)	C18—C19—C20—C21	−0.3 (2)
C18—O1—C9—C8	179.27 (14)	C19—C20—C21—C22	−0.6 (3)
C8—C9—C10—C11	1.6 (2)	C20—C21—C22—C23	0.7 (3)
O1—C9—C10—C11	−179.64 (14)	C19—C18—C23—C22	−1.0 (2)
C7—C6—C11—C10	−2.6 (2)	O1—C18—C23—C22	−177.33 (14)
N2—C6—C11—C10	175.98 (12)	C21—C22—C23—C18	0.1 (3)
C7—C6—C11—C15	177.79 (14)		

### Hydrogen-bond geometry (Å, º)

**Table:**

*D*—H···*A*	*D*—H	H···*A*	*D*···*A*	*D*—H···*A*
N2—H2*N*···S1^i^	0.88	2.53	3.3739 (15)	160

Symmetry code: (i) −*x*+1, −*y*+2, −*z*+1.
